# Immunogenicity of the Monovalent Omicron XBB.1.5-Adapted BNT162b2 COVID-19 Vaccine against XBB.1.5, BA.2.86, and JN.1 Sublineages: A Phase 2/3 Trial

**DOI:** 10.3390/vaccines12070734

**Published:** 2024-07-02

**Authors:** Juleen Gayed, Vishva Bangad, Xia Xu, Federico Mensa, Mark Cutler, Özlem Türeci, Uǧur Şahin, Kayvon Modjarrad, Kena A. Swanson, Annaliesa S. Anderson, Alejandra Gurtman, Nicholas Kitchin

**Affiliations:** 1 Vaccine Research and Development, Pfizer Ltd., Marlow International, Parkway, Marlow SL7 1YL, UK; nicholas.kitchin@pfizer.com; 2 Vaccine Research and Development, Pfizer Inc., Collegeville, PA 19426, USA; 3 BioNTech, 55131 Mainz, Germany; 4 Vaccine Research and Development, Pfizer Inc., Pearl River, NY 10965, USA

**Keywords:** BA.2.86, BNT162b2, booster, COVID-19, JN.1, Omicron, SARS-CoV-2, vaccine, variant-adapted, XBB.1.5

## Abstract

We report neutralization titer data against contemporary SARS-CoV-2 sublineages from an ongoing, phase 2/3, open-label, clinical trial of a single dose (30 μg) of an Omicron XBB.1.5-adapted BNT162b2 monovalent mRNA vaccine. The trial included healthy participants who had received at least three previous doses of an mRNA vaccine authorized in the United States, with the most recent authorized vaccine dose being a bivalent Omicron BA.4/BA.5-adapted vaccine given at least 150 days before the study vaccination. In this analysis, Omicron XBB.1.5, BA.2.86, and JN.1 serum neutralizing titers were assessed at baseline and at 1 month after vaccination. Analyses were conducted in a subset of participants who were at least 18 years of age (N = 40) and who had evidence of previous SARS-CoV-2 infection. Immunogenicity was also evaluated in a group of participants who received bivalent BA.4/BA.5-adapted BNT162b2 in another study (ClinicalTrials.gov Identifier: NCT05472038) and who were matched demographically to the participants in the current trial. In this analysis, monovalent XBB.1.5-adapted BNT162b2 vaccine elicited higher XBB.1.5, BA.2.86, and JN.1 neutralizing titers than those elicited by bivalent BA.4/BA.5-adapted BNT162b2. Overall geometric mean fold rises in neutralizing titers from baseline to 1 month after vaccination were higher among participants who received XBB.1.5-adapted BNT162b2 than those who received bivalent BA.4/BA.5-adapted BNT162b2 for XBB.1.5 (7.6 vs. 5.6), slightly higher for JN.1 (3.9 vs. 3.5), and similar for BA.2.86 (4.8 vs. 4.9). ClinicalTrials.gov Identifier: NCT05997290.

## 1. Introduction

More than 4 years after the COVID-19 pandemic was declared, COVID-19 continues to cause a significant health burden [1,2]. Vaccination against COVID-19 remains the best way to protect individuals against severe disease or death, and regular routine booster vaccinations are recommended in many age groups and populations [3,4]. Despite this, COVID-19 vaccine uptake for boosters remains poor; for example, in the United States (US) approximately 20% of all adults and less than half those 65 years of age and older are up to date with a booster vaccination [5].

The SARS-CoV-2 virus has evolved into more transmissible and immune-evasive variants; this has necessitated updating COVID-19 vaccine antigen compositions to more closely match circulating lineages [6]. The original BNT162b2 mRNA-based COVID-19 vaccine encoded the Wuhan-Hu-1 (ancestral) strain spike glycoprotein; bivalent vaccines were subsequently introduced, including the bivalent Omicron BA.4/BA.5-adapted BNT162b2, that targeted the ancestral strain plus Omicron BA.4/BA.5 components [7]. Currently, monovalent Omicron XBB.1.5-adapted COVID-19 vaccines, which only target Omicron XBB.1.5, are recommended. At the time of recommendation, Omicron XBB–descendent lineages were globally predominant [6]. However, SARS-CoV-2 continued to evolve, and the proportion of cases attributed to the Omicron JN.1 sublineage, which evolved from Omicron BA.2.86, has rapidly increased and is predominant globally as of April 2024 [8,9]. JN.1 shows higher immune evasion and diminished ACE2 binding affinity compared with its predecessors [9]. SARS-CoV-2 neutralizing titers have generally corresponded with vaccine effectiveness against symptomatic and severe disease, including hospitalization [10]. It is therefore important to evaluate neutralizing activity against currently circulating lineages following vaccination with recommended XBB.1.5-adapted vaccines.

Safety data and preliminary Omicron XBB.1.5, BA.2.86, and EG.5.1 immunogenicity responses 1 month after vaccination from a substudy of a clinical trial investigating XBB.1.5-adapted BNT162b2 in vaccine-experienced individuals have already been reported [7]. The aim of this brief report is to present additional immunogenicity data from the same study against Omicron XBB.1.5, BA.2.86, and JN.1. As the SARS-CoV-2 virus continues to evolve and sublineages may exhibit immune escape against recommended vaccines, these additional analyses were initiated to gain a better understanding of the immune responses elicited by XBB.1.5-adapted BNT162b2 against contemporaneous sublineages. 

## 2. Materials and Methods

### 2.1. Study Design and Participants

A full description of the methods, eligibility criteria, and ethical conduct of this study have been described previously [7]. Briefly, participants who were 12 years and older received a single dose (30 μg) of XBB.1.5-adapted BNT162b2 in a phase 2/3 study that is ongoing in the US (ClinicalTrials.gov Identifier: NCT05997290). This open-label study enrolled healthy individuals who had already received three or more doses of a US authorized mRNA COVID-19 vaccine. An inclusion criterion was that the most recent dose had to be a bivalent Omicron BA.4/BA.5-adapted COVID-19 vaccine administered ≥150 days before participants received the study vaccine. This report provides exploratory 1-month immunogenicity data from a subset of 40 participants aged 18 years and older. 

### 2.2. Objectives, Endpoints, and Assessments

The current analysis evaluated immunogenicity in the same variant neutralization subset as the previous analysis [7]. The randomly selected subset consisted of 40 participants (20 participants who were 18–55 years of age and 20 participants who were >55 years of age) who had evidence or a history of previous SARS-CoV-2 infection. Sera were collected at baseline and 1 month after vaccination and tested against Omicron XBB.1.5, BA.2.86, and JN.1 in a qualified assay (i.e., the fluorescent focus reduction neutralization test (FFRNT)), as described previously [11]. Briefly, the FFRNT is a high-throughput assay to determine neutralization titers from participant sera (i.e., maximum serum dilution inhibiting 50% of SARS-CoV-2). Geometric mean titers (GMTs) measured 1 month after vaccination and geometric mean fold rises (GMFRs) from before vaccination to 1 month after vaccination, and percentages of participants with seroresponses (i.e., at least a four-fold rise from baseline or at least four times the lower limit of quantitation (LLOQ) if baseline measurements were below the LLOQ) 1 month after vaccination are reported. At baseline and 1 month after vaccination, sera from a bivalent BA.4/BA.5-adapted BNT162b2 historical comparator group (N = 40; ClinicalTrials.gov Identifier: NCT05472038) were tested concurrently. The bivalent BA.4/BA.5-adapted BNT162b2 and monovalent XBB.1.5 vaccine participants were matched by SARS-CoV-2 infection status, age, and sex. For the bivalent BA.4/BA.5-adapted comparator group, participants had previously been administered three 30-μg doses of the original BNT162b2 vaccine; the last dose had to have been administered between 150 and 365 days before study vaccination with bivalent BA.4/BA.5-adapted BNT162b2. For both groups, testing of baseline samples and those taken 1 month after vaccination was conducted at the same time. 

Assessment of the safety and tolerability of XBB.1.5-adapted BNT162b2, which was the primary safety objective of this study, in the safety population of participants 12 years and older who received the study vaccine have been reported previously [7]. Briefly, reactogenicity events, which included local reactions (pain at the injection site, redness, swelling) and systemic events (fever, fatigue, headache, chills, vomiting, diarrhea, muscle pain, joint pain), were recorded by participants in an electronic diary for up to 7 days after vaccination. Adverse events and serious adverse events occurring within 1 month after vaccination were also reported. 

### 2.3. Statistical Analysis

Data are presented descriptively for participants overall and by age group. The GMTs and GMFRs were calculated through exponentiation of the mean and the mean of the difference between the two time points (at baseline and then 1 month after vaccination), respectively, of the log transformed assay results. The associated two-sided 95% CIs were determined using the Student *t* distribution. Seroresponse data are presented as percentages and corresponding 95% CIs calculated using the Clopper-Pearson method. The population studied for immunogenicity analyses was the evaluable immunogenicity population, which was comprised of individuals who were administered the study vaccine, had at least one valid immunogenicity result 1 month after vaccination visit (28 days to 42 days following vaccination), and who had no other major protocol deviations.

## 3. Results

### 3.1. Participants

In this analysis, among 40 participants who received XBB.1.5-adapted BNT162b2, 52.5% were female, 85.0% were White, 10.0% were Black, and 32.5% were of Latino or Hispanic ethnicity (Table A1). There were 20 participants in each of the two age groups (18–55 years and >55 years). The median age at vaccination was 35.5 (18–55-year-old group), and 69.5 years (>55-year-old group). The median time (and range) from the previous mRNA COVID-19 vaccination to study vaccination was 9.5 months (range, 2–12 months). Characteristics were generally similar for the 40 participants in the matched bivalent BA.4/BA.5-adapted cohort, apart from a lower percentage of participants with Latino or Hispanic ethnicity compared with the current study (17.5% vs. 32.5%) and a slightly longer mean interval from the previous COVID-19 mRNA vaccine dose compared with the current study (11.2 months vs. 9.1 months).

### 3.2. Immunogenicity

In the XBB.1.5-adapted BNT162b2 cohort, the evaluable immunogenicity population comprised 36 participants; four participants were not included in this population due to protocol deviations occurring at or before the 1 month visit after vaccination (one participant from the 18–55-year-old group; three participants from the >55-year-old group). In the matched bivalent BA.4/BA.5-adapted cohort there were 39 participants in the evaluable immunogenicity population; one participant was excluded from this population because the participant did not have at least one valid neutralization immunogenicity result obtained from 28 to 42 days of vaccination.

**Figure 1:**
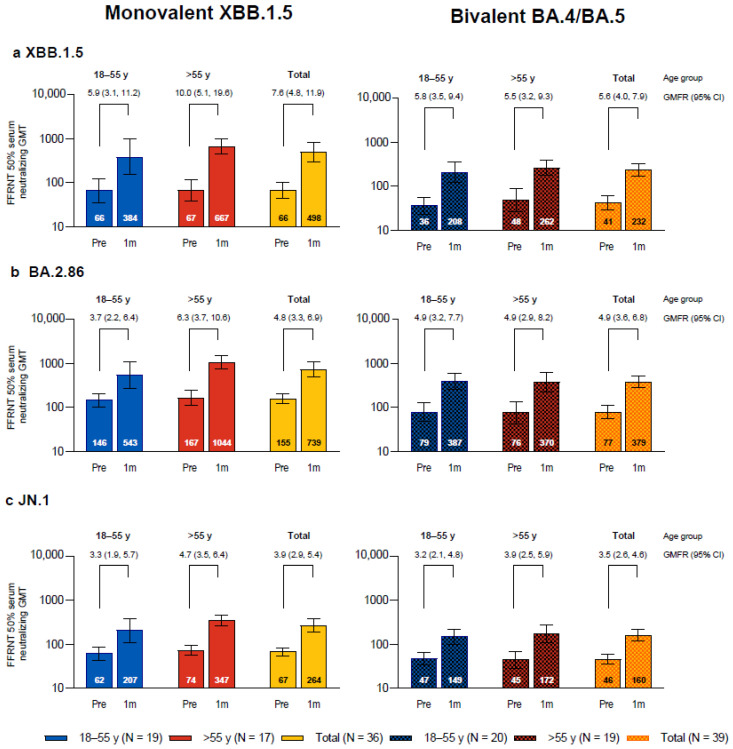
SARS-CoV-2 serum-neutralizing geometric mean titers (GMTs) with 95% CIs against Omicron XBB.1.5 (**a**), BA.2.86 (**b**), and JN.1 (**c**) before and 1 month after vaccination with monovalent XBB.1.5-adapted BNT162b2 or bivalent BA.4/BA.5-adapted BNT162b2. Geometric mean fold rises (GMFRs) and 95% CIs from before vaccination to 1 month after vaccination are also shown. Results that were less than the lower limit of quantitation (LLOQ) were set to 0.5 × LLOQ. 1m = 1 month after vaccination; FFRNT = fluorescent focus reduction neutralization test; Pre = before vaccination.

One month after vaccination with monovalent Omicron XBB.1.5-adapted BNT162b2, the SARS-CoV-2 FFRNT 50% neutralizing titers against the three sublineages tested, Omicron XBB.1.5, BA.2.86, and JN.1, increased compared to baseline levels and were each higher than those in the bivalent Omicron BA.4/BA.5-adapted BNT162b2 matched comparator group (Figure 1). In participants who received XBB.1.5-adapted BNT162b2, the GMTs at baseline were higher for Omicron BA.2.86 than for XBB.1.5 and JN.1; baseline GMTs were also slightly higher in participants who were >55 years of age compared to those who were 18−55 years of age for all three sublineages. GMTs at 1 month were higher for XBB.1.5 and BA.2.86 compared with JN.1. GMFRs from before to 1 month after study vaccination were higher for XBB.1.5 (GMFR overall, 7.6 (95% CI, 4.8, 11.9)) than for BA.2.86 (4.8 (3.3, 6.9)) and JN.1 (3.9 (2.9, 5.4)). Overall GMFRs from baseline to 1 month after vaccination were higher among the participants in this study who received XBB.1.5-adapted BNT162b2 than those in the matched comparator group who received bivalent BA.4/BA.5-adapted BNT162b2 for XBB.1.5 (7.6 vs. 5.6), slightly higher for JN.1 (3.9 vs. 3.5), and similar for BA.2.86 (4.8 vs. 4.9). In participants who received XBB.1.5-adapted BNT162b2, postvaccination titers in participants in the older group >55 years of age (GMT range, 347–1044) were generally higher compared to those from participants in the younger 18–55 years group (207–543).

**Figure 2:**
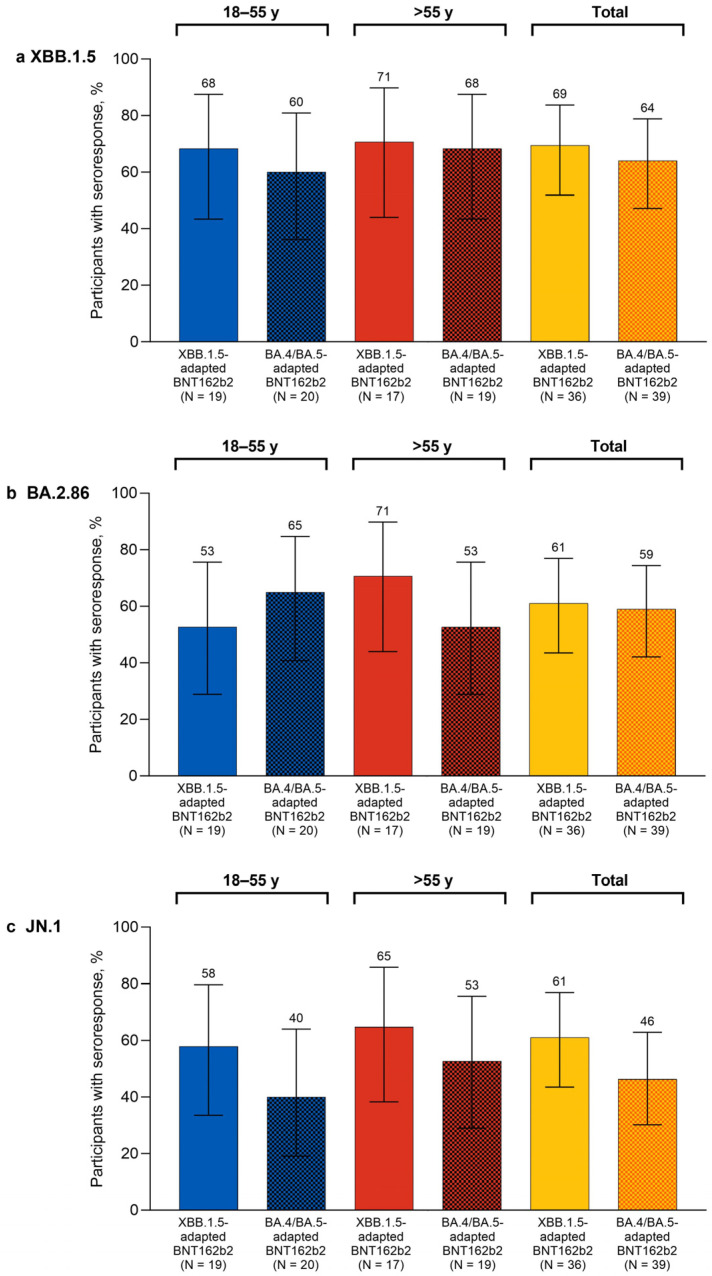
Percentages (95% CIs) of participants achieving seroresponses to Omicron XBB.1.5 (**a**), BA.2.86 (**b**), and JN.1 (**c**) 1 month after vaccination with monovalent XBB.1.5-adapted or bivalent BA.4/BA.5-adapted BNT162b2 30 μg. The definition of seroresponse was at least a 4-fold rise from before to 1 month after study vaccination in the SARS-CoV-2 FFRNT 50% serum neutralizing titers. For participants with a baseline measurement <LLOQ, seroresponse was defined as a postvaccination assay result of ≥4 × LLOQ. FFRNT = fluorescent focus reduction neutralization test; LLOQ = lower limit of quantitation.

One month after study vaccination, the percentages of participants overall with seroresponses in the XBB.1.5-adapted BNT162b2 group were higher than the corresponding percentages of participants in the bivalent BA.4/BA.5-adapted BNT162b2 matched comparator group for XBB.1.5 (69.4% vs. 64.1%), BA.2.86 (61.1% vs. 59.0%), and JN.1 (61.1% vs. 46.2%; Figure 2). The percentages of participants with seroresponses at 1 month after XBB.1.5-adapted BNT162b2 were generally similar in the 18–55-year-old and >55-year-old groups for Omicron XBB.1.5 (68.4% and 70.6%, respectively) and JN.1 (57.9% and 64.7%). The percentages of participants with seroresponses to Omicron BA.2.86 were slightly lower in the 18–55-year-old group (52.6%) than for the >55-year-old group (70.6%).

### 3.3. Safety

As described previously, local reactions occurring within 7 days after vaccination were of mild to moderate severity, and the most commonly reported local reaction was pain at the injection site [7]. Systemic events were mainly of mild to moderate severity and were most commonly fatigue and headache. Local reactions and systemic events occurred soon after vaccination (median onset of 1 to 2 days after vaccination) and were generally short lived (median duration of 1 to 3 days after vaccination). Fever (>38.9 °C to 40.0 °C) was reported in 0.5% of participants overall (*n* = 2) and occurred 1 day after vaccination, resolving 1 to 2 days after vaccination; no participant reported a fever >40.0 °C. Overall, 7.5% of participants in the overall population experienced adverse events within 1 month after vaccination. No adverse events leading to withdrawal, no vaccine-related serious adverse events, and no confirmed cases of myocarditis or pericarditis were reported through 1 month after study vaccination.

## 4. Discussion

This was an additional exploratory analysis from a phase 2/3 study of the monovalent Omicron XBB.1.5-adapted BNT162b2 vaccine in baseline-seropositive, vaccine-experienced adults ≥18 years of age. XBB.1.5-adapted BNT162b2 induced substantial neutralizing response increases against the three sublineages (Omicron XBB.1.5, BA.2.86, and JN.1). This subset is considered representative given that seropositivity in the general US population was over 96% by September 2022 [12]. In the previously published report from this study, XBB.1.5-adapted BNT162b2 elicited robust neutralizing responses to Omicron XBB.1.5 and EG.5.1 and slightly lower neutralizing responses to BA.2.86 [7]. Similarly, in this additional analysis, the immune response to BA.2.86 was preserved in a SARS-CoV-2 baseline-positive subset of participants, as previously reported in this subset [7], while the immune response to JN.1 was reduced. Overall JN.1 GMFRs from before to 1 month after receiving XBB.1.5-adapted BNT162b2 were approximately 1.9 and 1.2 times lower for Omicron XBB.1.5 and BA.2.86, respectively. These results are consistent with the higher immune escape potential of JN.1 compared to the earlier BA.2.86 and XBB.1.5 sublineages [9,13]. Overall GMTs, GMFRs, and seroresponses 1 month after vaccination were higher for XBB.1.5, BA.2.86, and JN.1 for the participants in the XBB.1.5-adapted BNT162b2 group than for those in the bivalent BA.4/BA.5-adapted BNT162b2 matched comparator group. The higher neutralizing titers obtained with XBB.1.5-adapted BNT162b2 versus bivalent BA.4/BA.5-adapted BNT162b2 for JN.1 is also consistent with immune escape of distant lineages to vaccine antigens; i.e., a closely matched vaccine antigen will likely elicit an improved neutralizing response and thus better protection than a mismatched antigen [14]. Although this analysis was neither powered nor designed to identify a difference between studies, these results are nevertheless encouraging.

Emerging immunogenicity and real-world effectiveness data for XBB.1.5-adapted vaccines are consistent with these observations. Other assessments of neutralizing antibody responses against BA.2.86 and JN.1 in those vaccinated with the XBB.1.5-adapted BNT162b2 or the XBB.1.5-adapted mRNA-1273 vaccine were found to be comparable or diminished compared to responses against XBB.1.5 but were nonetheless still potent [15,16,17]. These findings are also supported by emerging assessments of vaccine effectiveness against severe disease, including hospitalizations, urgent care or emergency department encounters, and medically attended outpatient COVID-19 following several months of use of XBB.1.5-adapted mRNA vaccines in immunization programs in Europe and North America [18,19,20,21]. Two of these studies, in the Netherlands and Denmark, were conducted mostly during XBB predominance [19,20], while observations from the other two studies, from the US and Canada, also included periods of JN.1 predominance [18,21]. Interestingly, a US study found that after approximately 4 months of use of XBB.1.5-adapted vaccines from September 2023 to January 2024, vaccine effectiveness against likely JN.1 lineages was 49% compared with 60% against non-JN.1 lineages with overlapping confidence intervals, thus providing support for the protection provided by XBB.1.5-adapted vaccines against a breadth of contemporary Omicron sublineages [22]. In contrast, another US study found that XBB.1.5-adapted vaccines were associated with a significantly lower risk of COVID-19 before JN.1 became dominant and a lower but nonsignificant risk after JN.1 predominance, with estimated vaccine effectiveness (95% CI) of 42% (32%, 51%) and 19% (−1%, 35%), for the two periods, respectively [23].

Limitations of the current analysis include the short follow-up through 1 month after vaccination. The exploratory analyses of immunogenicity parameters were also conducted in a small subset of predominantly White adults 18 years of age and older who were only from the US.

## 5. Conclusions

In conclusion, the additional immunogenicity data presented here continue to support administration of the currently recommended XBB.1.5-adapted BNT162b2 vaccine while also highlighting the potential need to periodically update COVID-19 vaccines to match circulating SARS-CoV-2 strains. 

## Data Availability

Upon request, and subject to review, Pfizer will provide the data that support the findings of this study. Subject to certain criteria, conditions, and exceptions, Pfizer may also provide access to the related individual de-identified participant data. See https://www.pfizer.com/science/clinical-trials/trial-data-and-results for more information.

## Appendix A

**Table A1:** Participant demographic and baseline clinical characteristics of the variant neutralization subset.

	Monovalent Omicron XBB.1.5-Adapted BNT162b2 30 μg			Bivalent Omicron BA.4/BA.5-Adapted BNT162b2 ^a^ 30 μg		
Characteristic	18–55 y (N = 20)	>55 y (N = 20)	Total (N = 40)	18–55 y (N = 20)	>55 y (N = 20)	Total (N = 40)
Sex, *n* (%)						
Male	12 (60.0)	7 (35.0)	19 (47.5)	12 (60.0)	7 (35.0)	19 (47.5)
Female	8 (40.0)	13 (65.0)	21 (52.5)	8 (40.0)	13 (65.0)	21 (52.5)
Race, *n* (%)						
White	18 (90.0)	16 (80.0)	34 (85.0)	17 (85.0)	16 (80.0)	33 (82.5)
Black	2 (10.0)	2 (10.0)	4 (10.0)	1 (5.0)	3 (15.0)	4 (10.0)
Other ^b^	0	2 (10)	2 (5.0)	2 (10)	1 (5.0)	3 (7.5)
Ethnicity, *n* (%)						
Hispanic/Latino	9 (45.0)	4 (20.0)	13 (32.5)	3 (15.0)	4 (20.0)	7 (17.5)
Age at vaccination, years						
Mean (standard deviation)	38.0 (10.04)	70.1 (6.51)	54.0 (18.30)	37.9 (9.97)	69.7 (5.95)	53.8 (18.00)
Median (range)	35.5 (25–55)	69.5 (56–82)	55.5 (25–82)	36.0 (25–54)	69.5 (56–81)	55.0 (25–81)
Baseline SARS-CoV-2 status, *n* (%)						
Positive ^c^	20 (100.0)	20 (100.0)	40 (100.0)	20 (100.0)	20 (100.0)	40 (100.0)
Time from last dose of mRNA COVID-19 vaccine to the study vaccination, months ^d^						
Mean (SD)	8.8 (1.98)	9.4 (2.53)	9.1 (2.26)	11.0 (1.41)	11.4 (1.16)	11.2 (1.29)
Median (range)	8.3 (5.8–12.0)	10.2 (2.0–11.9)	9.5 (2.0–12.0)	11.3 (7.4–12.8)	11.9 (8.5–12.6)	11.4 (7.4–12.8)
Body mass index, *n* (%)						
Underweight (<18.5 kg/m^2^)	0	1 (5.0)	1 (2.5)	0	0	0
Normal weight (≥18.5–24.9 kg/m^2^)	6 (30.0)	6 (30.0)	12 (30.0)	5 (25.0)	3 (15.0)	8 (20.0)
Overweight (≥25.0–29.9 kg/m^2^)	9 (45.0)	7 (35.0)	16 (40.0)	8 (40.0)	12 (60.0)	20 (50.0)
Obese (≥30.0 kg/m^2^)	5 (25.0)	6 (30.0)	11 (27.5)	7 (35.0)	5 (25.0)	12 (30.0)

^a^ Comparator group from another study (ClinicalTrials.gov Identifier: NCT05472038). ^b^ Includes American Indian or Alaska native, Asian, and multiracial. ^c^ Positive SARS-CoV-2 nucleoprotein–binding antibody result at baseline, positive nucleic acid amplification test result at baseline, or medical history of COVID-19. ^d^ Month was calculated as 28 days.
